# Genome Features of Probiotic Bifidobacteria Determining Their Strain-Specific Properties

**DOI:** 10.17691/stm2022.14.5.04

**Published:** 2022-09-29

**Authors:** A.G. Tochilina, I.V. Belova, T.N. Ilyicheva, V.Yu. Marchenko, V.A. Zhirnov, S.B. Molodtsova, A.V. Ikonnikov, I.V. Muhkina, A.S. Blagonravova, I.V. Soloveva

**Affiliations:** Senior Researcher, Laboratory of Human Microbiome and Means of Its Correction; Academician I.N. Blokhina Nizhny Novgorod Scientific Research Institute of Epidemiology and Microbiology of Rospotrebnadzor (Russian Federal Consumer Rights Protection and Human Health Control Service), 71 Malaya Yamskaya St., Nizhny Novgorod, 603950, Russia; Associate Professor, Department of Epidemiology, Microbiology and Evidence-Based Medicine; Privolzhsky Research Medical University, 10/1 Minin and Pozharsky Square, Nizhny Novgorod, 603005, Russia;; Leading Researcher, Laboratory of Human Microbiome and Means of Its Correction; Academician I.N. Blokhina Nizhny Novgorod Scientific Research Institute of Epidemiology and Microbiology of Rospotrebnadzor (Russian Federal Consumer Rights Protection and Human Health Control Service), 71 Malaya Yamskaya St., Nizhny Novgorod, 603950, Russia; Associate Professor, Department of Epidemiology, Microbiology and Evidence-Based Medicine; Privolzhsky Research Medical University, 10/1 Minin and Pozharsky Square, Nizhny Novgorod, 603005, Russia;; Leading Researcher, Department of Zoonotic Infections and Influenza; State Research Center of Virology and Biotechnology “Vector” of Rospotrebnadzor, Koltsovo, Novosibirsk Region, 630559, Russia; Leading Researcher, Department of Zoonotic Infections and Influenza; State Research Center of Virology and Biotechnology “Vector” of Rospotrebnadzor, Koltsovo, Novosibirsk Region, 630559, Russia; Researcher, Laboratory of Human Microbiome and Means of Its Correction; Academician I.N. Blokhina Nizhny Novgorod Scientific Research Institute of Epidemiology and Microbiology of Rospotrebnadzor (Russian Federal Consumer Rights Protection and Human Health Control Service), 71 Malaya Yamskaya St., Nizhny Novgorod, 603950, Russia;; Researcher, Laboratory of Human Microbiome and Means of Its Correction; Academician I.N. Blokhina Nizhny Novgorod Scientific Research Institute of Epidemiology and Microbiology of Rospotrebnadzor (Russian Federal Consumer Rights Protection and Human Health Control Service), 71 Malaya Yamskaya St., Nizhny Novgorod, 603950, Russia;; Research Assistant; Privolzhsky Research Medical University, 10/1 Minin and Pozharsky Square, Nizhny Novgorod, 603005, Russia;; Professor, Director of the Institute of Fundamental Medicine; Head of the Department of Normal Physiology named after N.Y. Belenkov; Privolzhsky Research Medical University, 10/1 Minin and Pozharsky Square, Nizhny Novgorod, 603005, Russia;; Head of the Department of Clinical Laboratory Diagnostics; Professor, Department of Epidemiology, Microbiology and Evidence-Based Medicine; Vice-Rector for Science; Privolzhsky Research Medical University, 10/1 Minin and Pozharsky Square, Nizhny Novgorod, 603005, Russia;; Associate Professor, Leading Researcher, Head of the Laboratory of Human Microbiome and Means of its Correction; Academician I.N. Blokhina Nizhny Novgorod Scientific Research Institute of Epidemiology and Microbiology of Rospotrebnadzor (Russian Federal Consumer Rights Protection and Human Health Control Service), 71 Malaya Yamskaya St., Nizhny Novgorod, 603950, Russia;

**Keywords:** *Bifidobacterium*, probiotics, antiviral activity, whole genome sequencing, influenza A virus

## Abstract

**The aim of the study** was to analyze the genome features of the probiotic strains *Bifidobacterium longum* 379, *Bifidobacterium bifidum* 1, and *Bifidobacterium bifidum* 791 and study their antiviral activity.

**Materials and Methods:**

Whole genome sequencing of three strains of bifidobacteria was performed on the MiSeq platform (Illumina Inc., USA). The genomes were annotated using the Prokka v. 1.11 utility and RAST genomic server. The individual genetic determinants were searched using the ResFinder 3.2, PathogenFinder, PlasmidFinder, RAST, and Bagel 4 software. The antiviral activity of the strains against influenza A viruses was studied using MDCK cells (Madin–Darby canine kidney cells), the epidemic strain of influenza A/Lipetsk/1V/2018 (H1N1 pdm09) (EPI_ISL_332798), the highly pathogenic avian influenza virus A/common gull/Saratov/1676/2018 (H5N6) strain (EPI_ISL_336925), and neutral red vital dye.

**Results:**

The genomes of all studied strains contained determinants responsible for utilization of carbohydrates of plant origin; the genes of key enzymes for the synthesis of tryptophan and folic acid are present in the genomes of *B. longum* 379 and *B. bifidum* 791. A feature of the *B. bifidum* 791 genome is the presence of determinants responsible for the synthesis of thermostable type I bacteriocins — flavucin and lasso peptide. The *B. bifidum* 791 strain was found to show pronounced antiviral activity against both the strains of influenza A, the supernatant of which suppressed viral replication *in vitro* up to a dilution of 1:8, and the cells inhibited viral reproduction up to a concentration of 6·106 CFU/ml.

**Conclusion:**

The analysis of complete genomes of *B. longum* 379, *B. bifidum* 1, and *B. bifidum* 791 showed features that determine their strain-specific properties, the findings on which were previously made empirically based on indirect signs. In the genomes of *B. longum* 379 and *B. bifidum* 791 strains, in contrast to *B. bifidum* 1 strain, key enzymes for the synthesis of tryptophan and folic acid were found. These substances have an impact on the human body in many ways, including having a thymoleptic effect (reducing emotional stress, irritability, anxiety, eliminating lethargy, apathy, melancholy, anxiety) and regulating cognitive activity. The presence of determinants responsible for the synthesis of thermostable type I bacteriocins in the genome of *B. bifidum* 791 strain determines its pronounced antiviral activity.

## Introduction

Bacteria of the *Bifidobacterium* genus have a number of unique properties and bring invaluable benefits to human health; therefore, they are traditionally used as strains producing probiotics [1].

One of the most important properties of bifidobacteria probiotics is their metabolic potential. According to the Academician A.M. Ugolev’s concept, there are two food flows coming from the intestines to other organs and tissues: the first one is the result of the absorption of products of enzymatic hydrolysis, and the second, no less significant, one is that of the absorption of products of bacterial hydrolysis (symbiotic digestion) [2]. As a result of hydrolysis by probiotic strains of dietary fiber, starch, oligosaccharides, etc., short-chain fatty acids are synthesized, which are valuable metabolites that have a complex beneficial effect on human health: energy supply of the epithelium, regulation of intestinal motility, strengthening of local immunity, etc. [2, 3].

Another essential property of probiotic strains is the ability to synthesize neurotransmitters and their precursors. For example, the normal microbiota is known to influence the concentration of serotonin, the most important neurotransmitter, both indirectly, by stopping local and systemic inflammatory processes, and directly, releasing its precursor, tryptophan. A lack of serotonin causes brain disturbance and mental changes such as increased anxiety, depression, and cognitive disorders [3, 4].

Currently, many researchers consider bifidobacteria as a therapeutic agent for the treatment and prevention of acute viral infections, the mechanisms of their antiviral action have been described, and the activity of bacteria against viruses has been shown to be a strain property [5-9].

Previously, the findings on the role and functions in the human body, biochemical and probiotic properties of bifidobacteria were made empirically, based on indirect signs. Currently, the use of whole genome sequencing with the help of modern services and methods of bioinformatics data processing makes it easy to prove the presence of genetic determinants presenting interest to the researchers, detect and describe the unique properties of a strain, and fully reveal its metabolic and probiotic potential. It should be noted that obtaining detailed information about the properties of specific probiotic strains is highly relevant in the light of implementation of personalized medicine, i.e. provides the selection of an individual probiotic, considering the health features and diagnosis of a patient.

**The aim of the study** was to analyze the genome features of probiotic strains *Bifidobacterium longum* 379, *Bifidobacterium bifidum* 1, and *Bifidobacterium bifidum* 791 and study their antiviral activity.

## Materials and Methods

The objects of the study were *B. bifidum* 1 (State Collection of Pathogenic Microorganisms, SCPM-Obolensk, No.900791), *B. bifidum* 791 (Bioresource Center Russian National Collection of Industrial Microorganisms (BRC VKPM), No.B-3300), *B. longum* 379 (BRC VKPM, B-2000) strains used for the production of probiotic medicines and foods.

Freeze-dried strains were reconstituted and a second generation of culture was prepared using a hydrolyzate-milk medium (Syntex, Russia), Bifidobacterium Agar medium (HiMedia, India), and GasPak EZ Anaerobe Gas Generating Pouch System with Indicator (BD, USA).

Species identification was performed using an Autoflex speed LRF time-of-flight MALDI mass spectrometer (Bruker Daltonics, Germany), sample preparation was performed according to the standard operating protocol “Extraction with formic acid” [10]. All measurements were carried out in a linear mode, detecting positive ions. Mass spectra were identified, recorded, processed, and analyzed using the BioTyper Rtc software [11]. The biochemical properties of the strains were confirmed using the API 20 A test system (BioMerieux, France).

Genomic DNA was isolated using a commercial QIAamp DNA Mini Kit (QIAGEN, Germany), fragmentation was performed using a Covaris E210 ultrasonic fragmentation system (Applied Biosystems, USA) according to the brand guidelines. The mixture was purified and fragments (200–700 bp) were selected using Agencourt AMPure beads (Beckman Coulter, USA) and NEBNext Sizing Buffer (New England Biolabs, USA). Libraries were prepared using the TrueSeq kit (Illumina Inc., USA), sequencing was performed on the MiSeq platform (Illumina Inc.). The initial nucleotide reads were processed using the Trimmomatic utility with standard parameters for Illumina. The processed reads were used for *de novo* genome assembly using the Spades, MIRA 4.0, Newbler 2.6 software.

The genomes were annotated using the Prokka v. 1.11 [12] utility and the RAST genomic server (https://rast.nmpdr.org). The determinants of antibiotic resistance and pathogenicity were searched using software products presented on the website of the Center for Genomic Epidemiology (www.cge.cbs.dk): ResFinder 3.2, PathogenFinder, and PlasmidFinder [13-15]. The Bagel 4 software was used to detect the genetic determinants responsible for the production of bacteriocins [16]. The key enzymes responsible for the synthesis of neurometabolites were searched using the RAST genomic server and scientific literature data [17-20].

The antiviral activity of strains against influenza A viruses was studied using cell suspensions of the third generation of each bifidobacteria culture, containing 10^8^ CFU/ml, grown in a hydrolysate-milk medium. The suspensions in a volume of 1 ml were centrifuged for 10 min at 3000 g; bacterial cells and supernatant liquid were taken to study the antiviral activity of the strains. The toxicity of cell suspensions, supernatants, and nutrient medium for MDCK cells (Madin–Darby canine kidney cells) was studied using the MTT assay [9].

To analyze the antiviral activity of the strains *in vitro*, we used MDCK cells, DMEM Gibco nutrient medium (Thermo Fisher Scientific, USA) with addition of 5% Gibco fetal bovine serum (Thermo Fisher Scientific) and antibiotics, as well as strains of influenza virus A/Lipetsk/1V/2018 (H1N1 pdm09) (EPI_ISL_332798) and A/common gull/ Saratov/1676/2018 (H5N6) (EPI_ISL_336925) obtained from the Collection of Microorganisms of the State Scientific Center of Virology and Biotechnology “Vector” of Rospotrebnadzor (Koltsovo, Russia). Vital dye of neutral red was used in the study [21].

The therapeutic index was calculated by dividing the 50% toxic dose by the 50% effective dose. To determine the statistical significance of differences in the values of the therapeutic index for different substances, the χ^2^ criterion was used. The calculation was carried out using the statistical software package Statistica 6.0. Differences were considered statistically significant at р<0.05.

## Results and Discussion

At the first stage of the work, the phenotypic features of the strains were studied by exploring their biochemical properties and protein mass spectra in order to select cultures intended for further sequencing.

After whole genome sequencing of the strains, the resulting reads were used to assemble *de novo* genomes using modern bioinformatics software. The genomes assembled in the contig format were registered in the international GenBank database. The main characteristics of the genomes of the studied strains are presented in the Table.

**Table:** Major genome features of the studied strains of the *Bifidobacterium species*

Strain	Number of contigs	Average coverage	Genome size (bp)	GC-content (%)	Number of CDS*	Number in GenBank
*B. longum* 379	24	150.0	2,387,620	60.2	1903	LKUQ00000000
*B. bifidum* 1	13	385.0	2,198,027	62.7	1521	NDXI00000000
*B. bifidum* 791	33	150.0	2,285,457	62.4	1769	LKUR00000000

* the sequences encoding proteins.

### Genome analysis of B. longum 379 strain

According to the data obtained using the PathogenFinder service, the genome of this strain does not contain pathogenicity determinants, and using the PlasmidFinder and ResFinder 3.2 software, it was found that the genome does not contain integrated plasmids or antibiotic resistance determinants. The data on the absence of antibiotic resistance determinants were confirmed using the RAST genomic server, though, other mechanisms of resistance were identified — molecular efflux pumps of the MATE family (GenBank: KYJ82530.1) and a cytoplasmic protein that protects the ribosome from tetracycline exposure — tetW (GenBank: KYJ81078.1).

**Figure 1.**
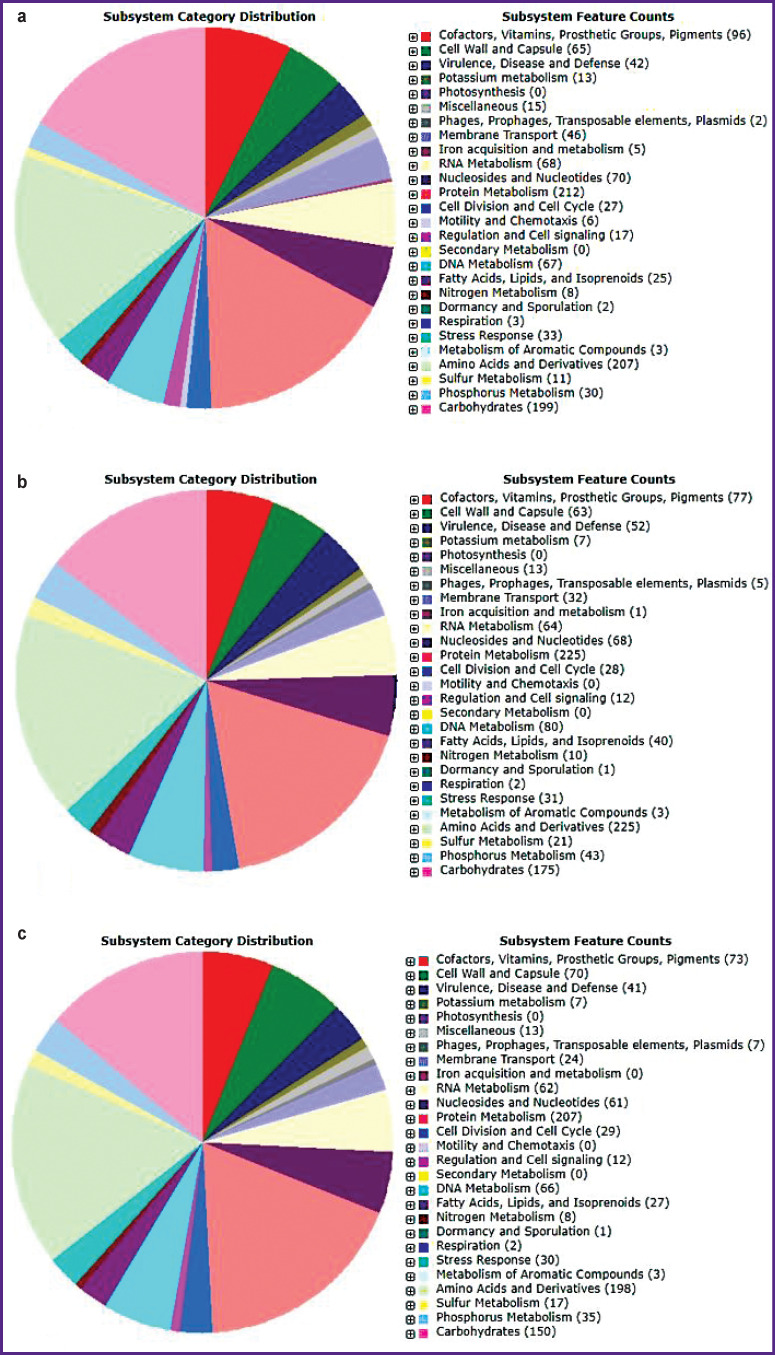
Functional annotation of the strains using the RAST genomic server: (a) *B. longum* 379; (b) *B. bifidum* 1; (c) *B. bifidum* 791

When analyzing the metabolic potential of the strain, it was found that the most widely represented subsystems are those of protein metabolism and sugar metabolism, consisting of 212 and 199 determinants, respectively (Figure 1 (a)).

The subsystem of sugar metabolism is represented by the determinants of the phosphoketolase pathway (fructose-6-phosphate phosphoketolase pathway), the products of which are lactic, acetic acids and ethanol, as well as determinants responsible for utilization of monosaccharides (xylose, ribose, arabinose), disaccharides (sucrose, maltose, lactose, raffinose and phospholigosaccharides), amino sugars, and starch. The amino sugar metabolism genes *NagA*, *NagB*, *NagK*, *NagR*, and *NagT* are located within the 8^th^ contig (NCBI: LKUQ01000023.1), and the locus responsible for starch metabolism is represented by the genes encoding the enzymes GAT (KYJ81114.1), GS (KYJ81082.1), GBr (KYJ82453.1), GP (KYJ83271.1), GdBr (starch degradation enzyme; KYJ82081.1), AMse (amylomaltase; KYJ82084.1), and MalE (maltose and maltodextrin transport protein; KYJ78457.1).

The operon for utilization of raffinose and fructooligosaccharides is represented by the determinants encoding proteins MsmR (regulatory protein; KYJ82093.1), MsmE, MsmF, MsmG (transport proteins; KYJ82142.1, KYJ82092.1, KYJ82141.1), SacA (KYJ78008.1), GtfA (sucrose phosphorylase), Aga (KYJ83465.1), BG (beta-glucosidase).

The determinants of exopolysaccharide synthesis: rhamnose synthesis genes located within the 5^th^ contig (NCBI: LKUQ01000020.1), capsular polysaccharides Wzb (tyrosine kinase; KYJ83195.1), Wzc (tyrosine phosphatase; KYJ83223.1), the determinants responsible for the formation of sortase-dependent pili — SrtA (sortase A; KYJ83477.1) and AP (KYJ83476.1), as well as lipoproteins — Lgt (KYJ83617.1) and LspA (KYJ77995.1) were found in the genome of the strain. The key enzymes of tryptophan synthesis — tryptophan synthase, α and β subunits (KYJ83618.1, KYJ83619.1) and folic acid synthesis — dihydropteroate synthase (KYJ81979.1) were also found in the strain genome.

### Genome analysis of B. bifidum 1 strain

According to the data obtained with the use of the PathogenFinder service, the genome of this strain does not contain pathogenicity-associated separate determinants or islands, and using the ResFinder 3.2 and PlasmidFinder services, it was found that the genome does not contain integrated plasmids or antibiotic resistance determinants. In the course of further analysis, using the RAST genomic server, the data on the absence of antibiotic resistance determinants were confirmed, and molecular efflux pumps of the MATE family (PDH98462.1) were found in the genome. Phenotypically, the presence of this molecular mechanism can be expressed in resistance to a number of antibacterial drugs.

It was revealed that the subsystems of metabolism of proteins (225 determinants) and sugars (175 determinants) are most widely represented in the strain (Figure 1 (b)). The sugar metabolism subsystem includes determinants of the phosphoketolase pathway, the products of which are lactic, acetic acids, and ethanol. The strain has a low ability to metabolize monosaccharides, though it is active against di-, oligosaccharides and amino sugars, those of chitin and N-acetylglucosamine. The operon of amino sugar utilization is represented by genes that determine the following enzymes: NagK (beta-galactoside-N-acetylhexosamine; GenBank: PDH98478.1), NagA (PDH97531.1), NagB (PDH98129.1), NagT PTS transport system (PDH97280.1), the regulatory protein NagR, as well as the enzymes CbsA (beta-hexominidase; PDH98222.1) and Aga (PDH97428.1). The genome also contains genes that determine starch metabolism enzymes: GAT, GS (PDH98335.1), GBr, GP (PDH97657.1), GdBr (PDH97486.1), AMse (amylomaltase).

Exopolysaccharide synthesis genes were not found, but there are determinants responsible for the formation of sortase-dependent pili SrtA (PDH97100.1, PDH97310.1), as well as cell wall lipoproteins Lgt (PDH98440.1) and LspA (PDH98074.1).

### Genome analysis of B. bifidum 791 strain

According to the data obtained using the PathogenFinder service, it was found that pathogenicity determinants are absent in the strain genome, and using the PlasmidFinder and ResFinder 3.2 software, it was found that the genome does not contain integrated plasmids or antibiotic resistance determinants. The detailed genome analysis using the RAST genomic server confirmed the absence of antibiotic resistance determinants, other mechanisms leading to phenotypically pronounced resistance to a number of antibiotics have been discovered, namely, molecular efflux pumps of the MATE family (KYJ84349.1, KYJ84414.1).

It was also found that the subsystems of protein metabolism and sugar metabolism, consisting of 207 and 150 genetic determinants, respectively, are widely represented (Figure 1 (c)). The sugar metabolism subsystem includes determinants of the phosphoketolase pathway, the products of which are lactic, acetic acids, and ethanol. In terms of its saccharolytic and other properties, *B. bifidum* 791 strain is close to *B. bifidum* 1 strain. Thus, the genome of *B. bifidum* 791 strain practically does not contain determinants of monosaccharide metabolism, however, there are genes responsible for the breakdown of more complex carbohydrates: di-, oligosaccharides, amino sugars, and starch.

The locus responsible for the utilization of amino sugars of the *B. bifidum* 791 strain is represented by the genes that determine the enzymes NagK (KYJ84330.1), NagA (KYJ85076.1), NagB (KYJ85077.1), the PTS transport systems NagT (KYJ85215.1, KYJ85216.1), regulatory protein NagR (KYJ85078.1), as well as CbsA (KYJ83728.1) and Aga (KYJ84485.1).

The genome contains determinants encoding enzymes of starch metabolism: GAT (KYJ83153.1), GS (KYJ84446.1), GBr (KYJ84933.1), GP (KYJ84691.1), GdBr (KYJ85154.1), AMse (KYJ84544.1) and the synthesis of exopolysaccharides, including, among other things, the rhamnose synthesis genes located within the 3^rd^ contig (NCBI: LKUR01000023.1), as well as the determinants responsible for the formation of sortase-dependent pili — SrtA (KYJ84870.1) and AP (KYJ84871.1) and cell wall lipoproteins — Lgt (KYJ84380.1) and LspA (KYJ85145.1). In addition, the key enzymes of tryptophan synthesis — tryptophan synthase, α and β subunits (KYJ84379.1, KYJ84378.1) and folic acid (dihydropteroate synthase (KYJ84132.1) were also found in the strain genome.

Within the 28^th^ contig (NCBI: LKUR01000021.1), the determinants responsible for the synthesis of the lasso peptide, a ribosomal-produced peptide exhibiting high antimicrobial and antiviral activity, and synthesis of bacteriocin flavucin, were found.

As a result of the analysis, it was confirmed that the *B. longum* 379, *B. bifidum* 1, and *B. bifidum* 791 strains are not pathogenic for humans, do not contain transmissible antibiotic resistance genes or integrated plasmids in the genome. Resistance to antibiotics of various classes is explained by the presence of molecular efflux pumps of the MATE family and the protective ribosomal protein TetW.

It has been established that all the strains are able to utilize di- and oligosaccharides, amino sugars, and starch. The products of their main metabolism are lactic and acetic acids*. B. longum* 379 and *B. bifidum* 791 contain exopolysaccharide synthesis determinants in their genomes, which are absent in *B. bifidum* 1. The genomes of all the strains contain determinants responsible for the formation of sortase-dependent pili and cell wall lipoproteins.

The ability of all studied strains to utilize carbohydrates of plant origin: amino sugars (that are widely distributed in nature and are part of cell membrane polysaccharides), fructooligosaccharides (vegetable sugars), raffinose (often found in seeds, root crops, vegetables), starch provides a competitive advantage for their existence as part of the intestinal microbiota and the implementation of probiotic properties (providing colonization resistance, antagonism against pathogenic and opportunistic microorganisms). On the other hand, the complex of active bacterial hydrolases ensures the most efficient assimilation of food by the macroorganism and active symbiotic digestion.

Valuable products of the active breakdown of carbohydrates by the studied strains of bifidobacteria are lactic (lactate) and acetic (acetate) acids, which are among the most important anions that provide slight acidification of the intestinal cavity, promote better absorption of electrolytes and inhibit the growth of pathogenic and opportunistic microorganisms. A high level of lactate contributes to the protrusion of macrophages (the penetration of their processes from the mucous membrane into the intestinal cavity) and determines the immunoregulatory properties of bifidobacteria. Acetate, a valuable short-chain fatty acid, solves important energy tasks when it is absorbed into the blood and gets into the cells of various organs and tissues. It also reduces the level of toxic metabolites and carcinogens, normalizes the motility of the gastrointestinal tract, and decreases the formation of ketones [2, 22].

The genomes of *B. bifidum* 791 and *B. longum* 379 strains were found to contain key enzymes for the synthesis of neurometabolites, those of tryptophan and folic acid. Tryptophan produced by bacteria and tryptamine formed as a result of its decarboxylation can be delivered with the bloodstream to the brain and perform the function of precursors of monoamine neurotransmitters in it [4]. Moreover, more than 90% of the total pool of the most important neurotransmitter serotonin in the human body is synthesized in the digestive tract as a result of the metabolization of tryptophan, supplied with food and produced by the microbiota [3]. A lack of serotonin causes pronounced brain disorders such as cognitive decline, increased anxiety and depression. Previously, researchers have obtained evidence of the ability of bifidobacteria to increase the level of tryptophan in the blood of laboratory animals, to alleviate their condition under stress, i.e. have a pronounced thymoleptic effect [3].

Folic acid (vitamin B_9_) is a water-soluble vitamin necessary for the growth and development of the circulatory and immune systems, its deficiency can cause megaloblastic anemia (macrocytosis), increases the risk of developing malignant tumors, and causes neural tube defects in the fetus during pregnancy. Folic acid deficiency is also typical of people suffering from depression [4]. Its main sources are food (vegetables, herbs, citrus, etc.) and human intestinal microbiota, i.e. the ability of the studied strains to synthesize it can be regarded as an important complementary mechanism for introducing this substance into the human body.

According to modern scientific data, among the main mechanisms of the inhibitory effect of probiotic bacteria on viruses, there is the production of active metabolites such as organic acids, bacteriocins, bactericidal substrates, etc., which lower the pH of the medium, prevent the adhesion of viral particles, and have a direct antiviral effect [23]. It has also been proven that exopolysaccharides of probiotic bacteria, sortase-dependent pili, as well as lipoproteins secreted by cell walls (LpAs), which physically prevent the interaction of viral particles with cell receptors [24]. Representatives of the *B. adolescentis*, *B. breve*, and *B. longum* species are known to exhibit antiviral activity [24-26].

In our study, all components of liquid cultures of bifidobacteria were non-toxic to MDCK cells; they did not reduce cell viability.

**Figure 2.**
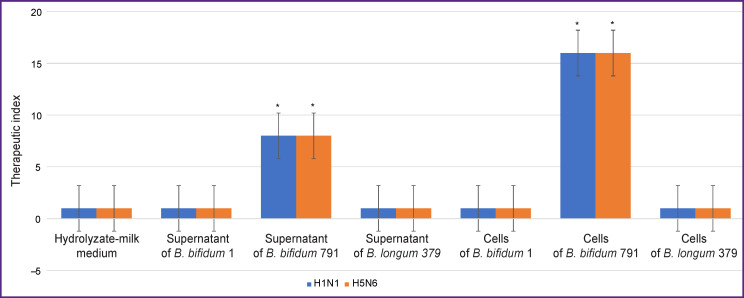
Therapeutic index of various components of the liquid culture of the *B. longum* 379, *B. bifidum* 1, and *B. bifidum* 791 bacteria in relation to the influenza virus strains A/Lipetsk/1V/2018 (H1N1 pdm09) (*blue*) and A/common gull/Saratov/1676/2018 (H5N6) (*orange*) The results are means ± standard deviations from three independent experiments; * p<0.05

Figure 2 presents the findings of the study of the antiviral activity of bifidobacteria against the epidemic strain of influenza virus A/Lipetsk/1V/2018 (H1N1 pdm09) and highly pathogenic strain A/common gull/ Saratov/1676/2018 (H5N6).

The supernatants and cells of *B. bifidum* 1 and *B. longum* 379 did not show a pronounced decrease in the cytopathic effect of the influenza virus on MDCK cells. Significant activity against both strains was found only in the supernatant obtained from centrifugation of 1 ml of *B. bifidum* 791 culture. In all dilutions up to 1:8, it suppressed virus replication *in vitro*. The cells of this strain inhibited virus reproduction up to a concentration of 6·10^6^ CFU/ml.

During the genome analysis of the *B. bifidum* 791 strain, it was found that it is capable of producing not only organic acids, secreted lipoproteins and exopolysaccharides, but also antibacterial peptides — flavucin and lasso peptide, belonging to the group of thermostable class I bacteriocins [16, 27]. Bacteriocins of this class have a wide spectrum of antiviral activity, are able to inhibit all stages of viral reproduction, block their receptors and enzymes, and prevent adsorption on eukaryotic cells [28-30]. We found that thermostable class I bacteriocins are the determining factor in the activity of probiotic strains of bifidobacteria against the epidemic influenza virus A/Lipetsk/1V/2018 (H1N1 pdm09) and highly pathogenic avian influenza virus A/common gull/Saratov/1676/2018 (H5N6).

## Conclusion

Using whole genome sequencing, new data on the strain-specific features of probiotic bifidobacteria were obtained. It has been established that the studied strains are able to utilize vegetable carbohydrates. The genomes of *B. longum* 379 and *B. bifidum* 791 strains, in contrast to *B. bifidum* 1 strain, contain key enzymes for the synthesis of tryptophan (precursor of monoamine neurotransmitters) and folic acid, the most important water-soluble vitamin. These substances have a multifaceted effect on the human body, including having a thymoleptic effect and regulating cognitive activity. A feature of the *B. bifidum* 791 genome is also the presence of determinants responsible for the synthesis of thermostable type I bacteriocins — flavucin and lasso-peptide, which determine the pronounced activity of this strain against the epidemic strain of influenza virus A/Lipetsk/1V/2018 (H1N1 pdm09) and highly pathogenic avian influenza virus A/common gull/ Saratov/1676/2018 (H5N6).
